# From Biomechanics to Bioinnovation: Emerging Applications of Piezoelectric Materials and Phenomena in Dentistry

**DOI:** 10.3390/biomedicines13112683

**Published:** 2025-10-31

**Authors:** Wen Kang, Yuehui Wang, Dan Zhao, Hongwei Wang, Sijing Xie, Lijia Pan

**Affiliations:** 1 Nanjing Stomatological Hospital, Affiliated Hospital of Medical School, Institute of Stomatology, Nanjing University, Nanjing 210008, China; 2 State Key Laboratory of Analytical Chemistry for Life Science, Medical School, Nanjing University, Nanjing 210093, China; hwang@nju.edu.cn; 3 Center for Translational Medicine and Jiangsu Key Laboratory of Molecular Medicine, Medical School, Nanjing University, Nanjing 210093, China; 4 Collaborative Innovation Center of Advanced Microstructures, School of Electronic Science and Engineering, Nanjing University, Nanjing 210093, China; yhwang0126@163.com

**Keywords:** teeth, piezoelectricity, piezoelectric biomaterials, dentistry

## Abstract

Teeth are the hardest organs in the human body. As mineralized structures, they possess a unique microstructure composed of orderly arranged piezoelectric materials such as hydroxyapatite crystals and collagen fibers. Teeth exhibit effective piezoelectric coefficients of approximately 1.2–1.6 pC/N. This inherent property enables teeth to function as natural piezoelectric sensors, converting routine mechanical stresses (e.g., chewing and biting forces, typically ranging from 22.4 to 68.3 kg) into localized electrical signals. This characteristic is of great importance in dentistry and materials science, offering new perspectives into a deeper understanding of the physiological functions and pathological mechanisms of teeth. Despite promising advances, challenges regarding the clinical translation, long-term stability, and biosafety of piezoelectric materials in the oral environment remain unresolved. This review highlights the biological functions of the piezoelectric properties of teeth, discusses recent applications and notable advancements of piezoelectric materials in dentistry, and outlines the challenges and research priorities for future clinical applications.

## 1. Introduction

Piezoelectricity refers to the property of certain materials to generate an electric charge in response to applied mechanical stress [1,2,3], or conversely, to undergo mechanical deformation when subjected to an electric field [4]. This phenomenon was first discovered in 1880 by brothers Jacques and Pierre Curie, and has since been widely applied in materials science [5], electronic engineering [6], and biomedical fields [7]. In recent years, with the rapid development of materials science and biomedicine, the study of piezoelectric effects in biological tissues and the application of piezoelectricity in biomedicine have gained increasing attention [8].

Teeth, as the crucial masticatory organs in the human body, are primarily composed of four tissues: enamel, dentin, cementum, and dental pulp [9]. Enamel and dentin not only exhibit excellent mechanical properties [10], but also demonstrate unique bioelectrical characteristics [11]. The average piezoelectric coefficient (d_33_) of enamel and dentin was determined to be 1.2 and 1.64 pC/N, respectively [12]. Although conventional piezoelectric ceramics, such as barium titanate (BaTiO_3_) (d_33_ = 190 pC/N), are widely recognized for their notable piezoelectric coefficient, their piezoelectricity originates from the intrinsic asymmetry of the crystal structure [13,14,15]. In contrast, piezoelectric phenomena in biological systems—particularly in teeth exhibit a distinct mechanism. The piezoelectricity in teeth originates primarily from the tissue’s inherent composite architecture, which is achieved through molecular-level coupling between hydroxyapatite (the inorganic phase) and collagen (the organic phase) [16]. In this structure, mechanical force induces ion displacement within hydroxyapatite crystals, generating weak electrical charges, while the embedded collagen fibers—owing to their flexibility and elasticity—effectively transmit and redistribute mechanical stresses, thereby synergistically modulating the overall piezoelectric response. This natural organic-inorganic nanocomposite structure enables teeth to produce endogenous bioelectrical signals under physiological masticatory forces, which in turn guide tissue repair and regeneration, whereas this biological function is absent in traditional single-phase piezoelectric ceramics.

Research on the piezoelectric properties of teeth contributes not only to a deeper understanding of their biomechanical and bioelectrical behaviors but also offers new insights and methods for dental tissue engineering, as well as the diagnosis and treatment of oral diseases. For instance, by mimicking the piezoelectric effects of dentin, the development of biocompatible piezoelectric materials holds promise for applications in teeth repair and regenerative medicine [17]. Furthermore, leveraging the piezoelectric properties of teeth could facilitate the design of novel oral sensors for real-time monitoring of oral health [18,19]. In dental clinics, teeth such as exfoliated primary teeth, impacted teeth extracted for pathological reasons, and teeth removed for orthodontic purposes are typically disposed of as medical waste through incineration by waste management companies [20,21]. Recycling these dental biowastes for energy harvesting presents a promising opportunity to transform biological waste into a source of green energy. For example, Yin et al. fabricated a nanogenerator from piezoelectric teeth enamel and dentin. Under an external mechanical force of 60 N, the device generated an open-circuit voltage of 0.9 V and successfully illuminated a light-emitting diode [12]. However, the potential safety risks should be taken into consideration.

**Figure 1:**
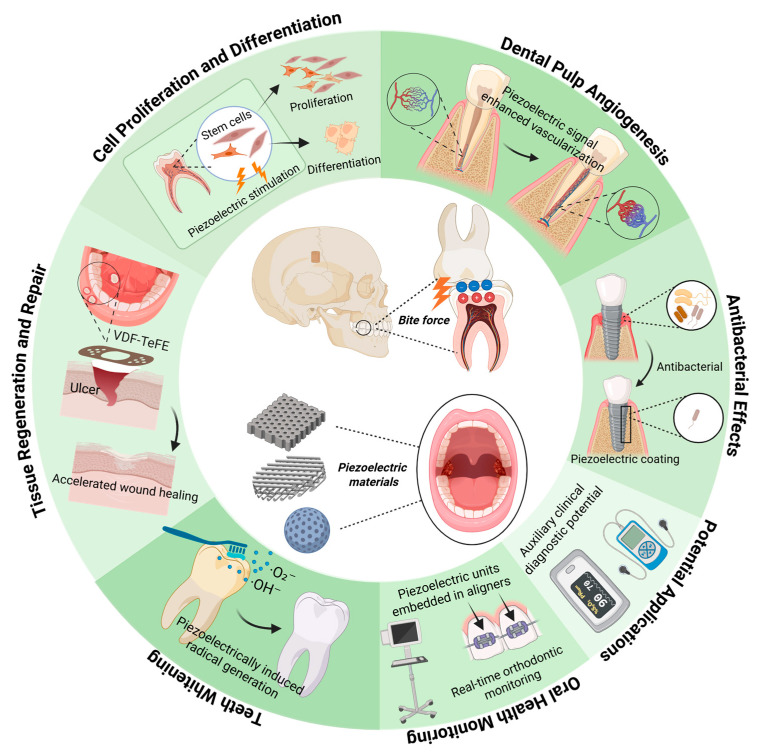
The role of dental piezoelectricity and advancements in piezoelectric materials for dentistry. Created in BioRender. Zhao, C. (2025) https://BioRender.com/rgx3upe.

This review summarizes the piezoelectric properties of enamel and dentin, discusses the biological functions of piezoelectric effects in teeth, and highlights the applications and challenges of piezoelectric materials in the field of dentistry, thereby providing a theoretical foundation for the development of new dental materials and treatment technologies (Figure 1).

## 2. Piezoelectricity in Teeth

### 2.1. Fundamental Principles of the Piezoelectric Effect

The piezoelectric effect refers to the phenomenon wherein certain non-centrosymmetric materials generate internal polarization when subjected to mechanical stress along specific directions, resulting in the accumulation of opposite charges on their opposing surfaces [22]. This effect originates from the unique crystal structure of piezoelectric materials [23,24,25]. In such crystals, atoms or ions are arranged in a non-centrosymmetric manner. Under external force, the crystal structure deforms, causing displacement of atoms or ions. This shifts the centers of positive and negative charges, breaking symmetry and inducing electric polarization. As a result, charges accumulate on the crystal surfaces, forming the direct piezoelectric effect [26]. Conversely, when an electric field is applied, it exerts a force on the charges within the crystal, causing atomic or ionic displacement and resulting in mechanical deformation—known as the inverse piezoelectric effect [27,28]. In contrast, crystals of non-piezoelectric materials possess centrosymmetric atomic or ionic arrangements. Under external force, the internal charge distribution remains unchanged, and no piezoelectric effect occurs [29,30].

### 2.2. The Piezoelectric Properties of Teeth

In 1957, the pioneering study by E. Fukada and colleagues first identified piezoelectric properties in bone tissue [31,32]. Owing to the structural resemblance between dental and osseous tissues, considerable research efforts have since been devoted to examining whether similar piezoelectric behavior occurs in dental hard tissues. This research trajectory culminated in the seminal work of M. Braden et al. in 1966, which provided definitive evidence of piezoelectricity in human teeth [33]. Dental hard tissues comprise three principal constituents: enamel, dentin, and cementum [34]. The inorganic composition of these tissues predominantly consists of hydroxyapatite (Ca_10_(PO_4_)_6_(OH)_2_). Enamel, the hardest substance in the human body, forms the outermost layer of the teeth and consists of approximately 96% hydroxyapatite by weight, with collagen and water accounting for the remaining 4% [35]. Dentin, located beneath the enamel, contains about 70% hydroxyapatite and 30% collagen and water [36].

**Figure 2:**
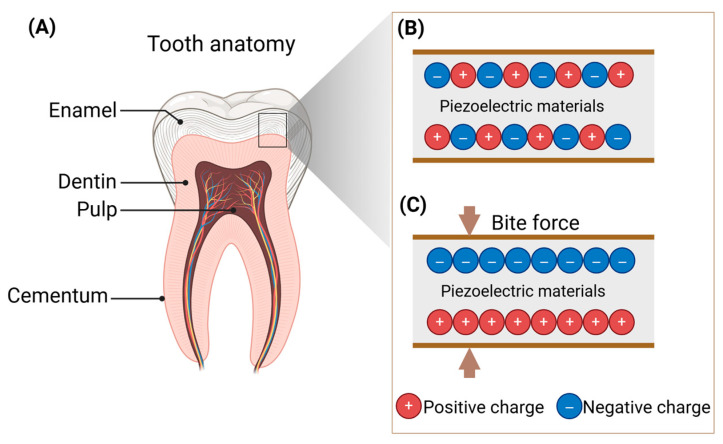
The anatomy of teeth and the piezoelectric principle. (**A**) The anatomy of teeth. (**B**,**C**) Teeth generating electrical charges under biting force. Created in BioRender. Zhao, C. (2025) https://BioRender.com/u928znc.

When subjected to masticatory forces, mechanical deformation induces relative displacements of calcium (Ca^2+^), phosphate (PO_4_^3−^), and hydroxide (OH^−^) ions within the hydroxyapatite crystal lattice, resulting in asymmetric charge distribution and consequent piezoelectric polarization (Figure 2). Collagen fibrils, as a key organic component in dental hard tissues, exhibits high flexibility and elasticity. It interweaves with hydroxyapatite crystals to form an organic-inorganic composite structure, which not only enhances the mechanical properties of teeth but also modulates their piezoelectric behavior by altering stress transmission and deformation mechanisms at the crystalline level. Notably, investigations by A.A. Marino et al. have revealed a positive correlation between piezoelectric coefficients and organic content in dental tissues [37]. This finding explains the superior piezoelectric performance observed in dentin (d_33_ = 1.64 pC/N; 30% organic content) compared to enamel (d_33_ = 1.2 pC/N; 4% organic content) [12]. Compositional analysis shows that cementum contains approximately 55% organic matter and water [38], so it may exhibit a stronger piezoelectric effect than dentin or enamel. As expected, further stress-charge measurements revealed that the cementum and dentin from sperm whale teeth exhibit piezoelectric coefficients of 0.028 and 0.027 pC/N, respectively [37]. However, whether this conclusion holds for human teeth remains to be experimentally verified through dedicated studies on their piezoelectric properties. Studies have also reported variations in piezoelectric properties across different regions of the enamel within the same tooth [39,40]. This phenomenon is related to factors such as localized tooth microstructure, chemical composition, and conditions during dental development [41].

### 2.3. Role of Piezoelectricity in Teeth

#### 2.3.1. Effects on Cell Proliferation and Differentiation

Teeth are subjected to various mechanical stresses during mastication and occlusal activities. These stresses induce endogenous piezoelectric signals, which can be perceived and transduced by cells—the fundamental structural and functional units of organisms—leading to alterations in a range of biological behaviors [42,43,44].

Ion channels serve as critical molecular sensors for piezoelectric signals. For example, Piezo channels undergo conformational changes under piezoelectric stimulation, facilitating transmembrane ion flow and generating intracellular electrical signals. Piezo1 and Piezo2 channels are expressed in diverse cell types, including periodontal ligament stem cells, dental pulp stem cells (DPSCs) [45], and osteoblasts [46], where they play essential roles in cellular mechanotransduction and electrochemical signal sensing. For instance, in osteoblasts, Piezo1 detects extracellular mechanical stimuli, converts them into electrical signals, and activates downstream signaling pathways to regulate proliferation and differentiation [46].

Dental pulp stem cells exhibit notable mechanosensitivity. In vitro studies have shown that mechanical stimulation significantly enhances their proliferation [45,47,48]. Exposure to piezoelectric-mimicking electric fields further promotes the proliferation and differentiation of these cells [49,50]. Mechanistic investigations reveal that piezoelectric signals activate intracellular pathways such as extracellular signal-regulated kinase (ERK), mitogen-activated protein kinase (MAPK), and cyclic adenosine monophosphate/protein kinase A (cAMP/PKA), which facilitate cell cycle progression and ultimately regulate cellular proliferation and differentiation [51].

Previous studies have shown that culturing mesenchymal stem cells on 3D-printed hydroxyapatite scaffolds markedly enhances osteogenic differentiation [52,53]. In addition, according to the report by Mirzaei et al., the piezoelectric composite scaffold made by electrospinning polyvinylidene fluoride (PVDF) and polyaniline can induce the osteogenic differentiation of human dental pulp stem cells under the action of pulsed electromagnetic fields [54]. It is reasonable to believe that, within the dental microenvironment, the occlusal force activates the piezoelectric properties of hydroxyapatite, while the weak electrical stimulation in turn modulates the differentiation of dental pulp stem cells. Therefore, piezoelectric-film-based biomimetic electrophysiology can be exploited to promote the regeneration of natural dental hard tissues, which carries far-reaching clinical implications.

**Figure 3:**
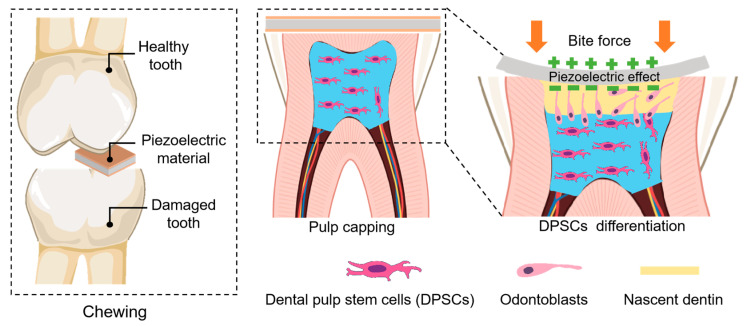
Schematic diagram illustrating the relationship among biomechanics, the piezoelectric effect, and dental tissue regeneration pathways. Created in BioRender. Zhao, C. (2025) https://BioRender.com/dspmppa.

From a clinical perspective, the cellular mechanisms regulated by piezoelectricity open new avenues for regenerative dentistry. For instance, the ability of piezoelectric signals to promote dental pulp stem cell proliferation and odontoblastic differentiation suggests a promising therapeutic approach for pulp regeneration in early pulpitis [55,56]. This could translate into applying piezoelectric-mimicking electrical stimulation during direct pulp capping or pulpotomy to enhance residual pulp tissue regeneration and improve vital pulp preservation rates (Figure 3). Similarly, for periodontal bone loss, piezoelectric biomaterials could be developed to harness physiological bite forces, generating endogenous electrical signals that promote osteogenic differentiation of mesenchymal stem cells and accelerate bone regeneration. However, the biological safety and long-term stability of these piezoelectric biomaterials also need to be carefully considered before they can progress to clinical trials and commercial use.

#### 2.3.2. Effects on Angiogenesis in Dental Pulp

The dental pulp is a highly vascularized tissue, and angiogenesis is crucial for pulp regeneration engineering. Electrical stimulation has been reported to promote vascularization in dental pulp by upregulating the expression of angiogenic factors such as vascular endothelial growth factor (VEGF) and fibroblast growth factor (FGF), and activating the phosphatidylinositol 3-kinase/protein kinase B (PI3K/Akt) signaling pathway in endothelial cells [57,58].

Clinically, the angiogenic potential holds particular relevance for pulp revascularization in young permanent teeth with immature roots [59]. Building on evidence that piezoelectric-mimicking electrical stimulation upregulates VEGF and FGF expression, clinicians could incorporate targeted electrical stimulation into revascularization protocols. This approach may enhance neovascularization within the necrotic pulp chamber, thereby facilitating root elongation and apical closure. Furthermore, dental pulp stem cell transplantation has emerged as a novel strategy for pulp regeneration following pulpitis and dental trauma [60]. For patients with severe pulp damage, pre-treating dental pulp stem cell-seeded scaffolds with piezoelectric signals before transplantation could enhance vascularization within the regenerated pulp tissue. However, several key challenges require resolution, including the precise optimization of electrical stimulation parameters and ensuring the long-term maintenance of neovascularization within the complex and dynamic pulp microenvironment.

## 3. Application of Piezoelectric Materials in Dentistry

### 3.1. Tissue Regeneration and Repair

Electric fields play a regulatory role in numerous physiological processes in the human body [44]. Piezoelectric materials, capable of generating electric fields under mechanical stress, offer unique advantages in tissue regeneration and repair. Certain cell types within dental tissues, such as odontoblasts, exhibit sensitivity to electric fields [61]. Teeth mineralization relies on the involvement of odontoblasts (responsible for dentin formation) and ameloblasts (responsible for enamel formation) [62,63], and electric fields are known to influence the function and behavior of these cells. Li et al. developed a strontium-doped piezoelectric biofilm to induce dentin mineralization regeneration. When applied to dentin defects, this biofilm generates a micro-electric environment under masticatory stress, providing not only surface potential but also releasing strontium ions. This successfully recruits dental pulp stem cells and promotes their differentiation into odontoblasts, upregulating the expression of mineralization-related proteins such as runt-related transcription factor 2 (Runx2), dentin sialophosphoprotein (DSPP), and dentin matrix protein 1 (DMP-1), thereby enhancing cellular mineralization capacity and facilitating endogenous dentin regeneration [64]. BaTiO_3_ particles have been incorporated into dental composite restorations due to their piezoelectric properties. Under mechanical stimulation, the resulting electric field promotes remineralization and self-repair of dental hard tissues, strengthens the bond between the restorative material and teeth structure, and enhances the repair of defective teeth tissues [65]. Piezoelectric hydrogels can modulate energy metabolism and promote ATP synthesis, initiating the osteogenic differentiation of periodontal ligament stem cells. They also polarize pro-inflammatory M1 macrophages toward the anti-inflammatory M2 phenotype, thereby remodeling the immune microenvironment to be anti-inflammatory and pro-regenerative, which facilitates periodontal tissue regeneration [66]. Under ultrasound assistance, piezoelectric nylon-11 nanoparticles significantly enhance the osteogenic differentiation of dental pulp stem cells and repair alveolar bone defects [67]. During oral mucosal wound healing, a copolymer of vinylidene fluoride and tetrafluoroethylene (VDF-TeFE), used as a piezoelectric material, can be applied to damaged mucosal sites to promote cells growth and repair, accelerate the regeneration of oral mucosal tissues [68,69], restore fibrous components, and reduce inflammatory responses during healing [70].

### 3.2. Antibacterial Effects

Piezoelectric materials exhibit inherent antibacterial properties through electrical charges generated under mechanical stimulation, offering a promising alternative that circumvents concerns regarding bacterial resistance [71,72]. A common cause of dental implant failure is peri-implant infection [73]. To address this, Sun et al. developed a novel implant material featuring a multifunctional three-dimensional piezoelectric coating on the titanium surface, composed of hierarchical TiO_2_ nanotubes (NTs) and an electrospun PVDF nanofiber layer. This design enables the injection of positive charges into the NT layer via electric field induction, forming charge traps. The well-tuned pore size and electrostatic interactions of the coating facilitate bacterial movement through the nanofiber layer toward the NTs, where trapped bacteria are effectively killed by the positive charges, resulting in significant antibacterial activity [74]. Similarly, Xu et al. integrated metal/piezoelectric nanostructures onto implant surfaces, which under ultrasonic activation effectively inhibit the attachment of *Staphylococcus aureus*, thereby reducing the risk of post-surgical infections [75]. The antibacterial mechanisms of piezoelectric materials are primarily classified into two distinct categories based on their reactive oxygen species (*ROS*) generation pathways: piezocatalysis and photocatalysis [76,77,78]. The former describes *ROS* production driven by mechanical energy, where material deformation creates surface charges that directly catalyze redox reactions. As a representative piezocatalytic strategy, the incorporation of BaTiO_3_ nanoparticles into denture generates *ROS* through surface charge stimulation under masticatory force, constituting a light-independent antibacterial mechanism [79]. Dental caries is a bacteria-driven infectious disease [80]. The incorporation of BaTiO_3_ into dental composite resins—as a restorative material for caries—not only suppresses microbial growth but also promotes teeth remineralization [65]. Further studies have demonstrated that adding zinc-doped mesoporous silica nanoparticles (Zn-MSNs) or functionalized methyl methacrylate (K18-MMA) and glass filler (K18-Filler) into composites can effectively inhibit the progression of dental caries [81,82]. Dental plaque is a primary etiological factor in periodontal diseases, which can progress to alveolar bone resorption, teeth mobility, and eventual teeth loss [83,84]. In response, Lina Roldan et al. developed an injectable piezoelectric hydrogel (PiezoGEL) by combining gelatin methacryloyl (GelMA) with biocompatible BaTiO_3_ piezoelectric fillers. Under masticatory forces, this hydrogel generates electrical charges that exert antibacterial effects while simultaneously promoting the regeneration of bone tissue [85].

The biosafety of BaTiO_3_ for oral applications requires careful evaluation, particularly regarding the potential leaching of Ba^2+^ in oral environments. In vitro studies confirmed that BaTiO_3_ (across concentrations of 1–60% wt/wt) maintained normal metabolic activity and viability of dental pulp stem cells. At 100 mg filler concentration, cell proliferation increased significantly, accompanied by an 80% rise in viable dental pulp stem cell numbers [65]. Further supporting these findings, BaTiO_3_/hydroxyapatite scaffolds exhibited no detectable cytotoxicity, with dental pulp stem cells showing elevated cell density on the surface of scaffolds during extended culture [86]. Hua et al. demonstrated that BaTiO_3_/hydroxyapatite nanocomposites sustained mesenchymal stem cells viability substantially above the 100% negative control threshold, reaching a peak of 233.5% after 72 h [87]. Collectively, these findings affirm the exceptional cytocompatibility, low cytotoxicity, and promising biomedical potential of BaTiO_3_-based biomaterials. Nevertheless, their clinical translation faces considerable challenges. Current evidences remain largely limited to short-term in vitro studies, with long-term safety data under clinically relevant conditions still lacking. Furthermore, regulatory approval from agencies such as the FDA will require comprehensive biosafety evaluation in accordance with ISO 10993 standards, a necessary prerequisite before any clinical application can proceed [88].

### 3.3. Teeth Whitening

With growing emphasis on oral aesthetics, teeth whitening has emerged as a prominent aspect of oral healthcare [89]. Most commercially available medical teeth-whitening products currently employ peroxides—such as hydrogen peroxide or carbamide peroxide—at concentrations exceeding 30% as active agents [90]. However, these high-concentration peroxides can induce irreversible enamel damage and even pulpitis during the whitening process [91]. Consequently, the development of efficient and safe whitening technologies has become a key research focus in oral medicine. Piezoelectric materials have demonstrated unique advantages for teeth whitening applications, primarily through piezoelectric catalytic effects [92,93]. Wang et al. integrated piezoelectric BaTiO_3_ with oral care practices by utilizing mechanical vibrations from toothbrushing to activate the material’s piezoelectric response. The resulting charge release interacts with water molecules, continuously generating reactive free radicals (·OH^−^ and·O_2_^−^) capable of oxidizing and breaking down pigment macromolecules into small colorless compounds, thereby achieving effective whitening [90]. In addition, compared with the widely used hydrogen-peroxide-based clinical whitening agents, piezocatalytic teeth whitening with BaTiO_3_ nanoparticles is non-destructive to enamel, biocompatible, and cytotoxicity-free [90]. In another study, He et al. developed a Z-scheme g-C_3_N_4__−__x_/Bi_2_O_3__−__y_ heterostructure that synergistically combines photocatalysis and piezocatalysis, enabling efficient and non-destructive teeth whitening with additional antibacterial benefits [94]. These advances introduce a novel and safe paradigm for aesthetic dentistry, offering an effective peroxide-free alternative that is both user-friendly and cost-effective. They demonstrate promising application potential. Future research should thoroughly assess the clinical feasibility of piezoelectric teeth-whitening platforms and translate these findings into practical products.

### 3.4. Oral Health Monitoring

Piezoelectric materials facilitate the conversion of mechanical energy into electrical signals [95,96,97,98]. The deployment of highly sensitive piezoelectric sensors on teeth surfaces or within the oral cavity allows real-time monitoring of force-induced charge variations. These signals contain rich physiological information related to bite force, mastication patterns, and teeth mobility, enabling continuous assessment of oral health status [99]. Such capabilities provide valuable support for early diagnosis and prevention of oral diseases. For instance, piezoelectric pressure sensors can track occlusal forces in real time. This provides objective data for evaluating masticatory performance and occlusal balance, which guides the design and optimization of dental prostheses [100]. Tan et al. prepared a high-performance dopamine (DA)/polyvinyl alcohol (PVA)/glycine thin film by a simple one-step method, achieving a piezoelectric coefficient of 10.8 pC/N. By further encapsulating the film into a waterproof force sensor, four occlusal contact patterns were successfully monitored, offering valuable insights for evaluating the occlusal contact patterns during restorative or orthodontic treatments [99]. Bruxism is a common oral parafunctional disorder characterized by involuntary clenching or grinding of teeth during sleep. This condition can lead to a range of clinical complications, including teeth wear, orofacial pain, and headaches [101]. Kazuyoshi Baba et al. embedded piezoelectric films on the occlusal surfaces of maxillary teeth to transduce biting force into electrical signals, allowing quantitative monitoring of bruxism episodes and supporting clinical diagnosis and management. The force-sensing device demonstrates accuracy in recording sleep bruxism that is no less than that of single-channel electromyography-based recordings [18,102]. Future studies could focus on improving wearing comfort to enhance patient compliance. Additionally, Wang et al. developed a personalized bite-activated orthodontic system incorporating flexible piezoelectric units within dental aligners [19]. This device harnesses occlusal mechanical energy to generate pulsed electric fields, which enhance osteoblasts and osteoclasts activity, thereby optimizing bone remodeling and accelerating teeth movement. Within an 18-day intervention, young and old Sprague-Dawley rats showed significantly improved orthodontic outcomes, with teeth movement increasing by 34% and 164%, respectively, compared to the mechanical orthodontic aligners. The system also permits real-time monitoring of teeth displacement rates, offering a theranostic approach for orthodontic treatment across diverse age groups. In addition, polypropylene piezoelectric films are inherently flexible and lightweight, allowing them to conform seamlessly to teeth surfaces while offering excellent mechanical robustness and biocompatibility—making them well-suited for long-term patient wear.

### 3.5. Potential for Diagnosis and Treatment of Oral Diseases

The innate piezoelectric property of teeth suggests its potential use as a diagnostic indicator in early-stage oral diseases. Pathological changes such as dental caries or cracks alter the microstructure and mechanical properties of teeth tissues [103,104], thereby influencing their piezoelectric responses. These variations can be captured via piezoelectric sensors. For example, El-Sharkawy et al. used spectral analysis to examine the responses of teeth samples, which were accurately detected by piezoelectric transducers, thereby categorizing a tooth as carious or normal [105]. In addition, the injectable PiezoGEL developed by Rolland et al. can effectively reduce periodontal inflammation and promote bone tissue regeneration, thus having good application prospects in the treatment of periodontal disease [85]. Although effective, the biological safety and metabolic fate of piezoelectric materials after they enter the human body are key issues that must be addressed before clinical use. In particular, some inorganic piezoelectric materials contain metal ions that may be toxic [106]. Natural piezoelectric materials such as amino acids, owing to their biocompatibility, high performance, and low cost, can be regarded as priority research targets [107]. Furthermore, establishing a comprehensive database of piezoelectric signal characteristics from both healthy and diseased teeth, combined with advanced signal processing and artificial intelligence algorithms, can enable accurate interpretation of real-time sensor data. This approach may facilitate early detection of dental abnormalities, supporting timely intervention and prevention of disease progression.

Based on the current progress, the applications of piezoelectric materials in dentistry and the corresponding in vitro, in vivo, and clinical studies are summarized in Table 1 and Table 2, respectively.

## 4. Conclusions and Outlook

The discovery of the piezoelectric properties in teeth holds significant theoretical and practical implications. Theoretically, it provides a novel mechanism for interpreting the physiological functions of teeth. While traditional views have emphasized the role of mechanical structures in mastication and occlusion, the existence of piezoelectricity suggests that teeth also generate electrical signals under mechanical stress. These signals may contribute to the perception and regulation of chewing forces, as well as to the modulation of physiological activities in dental pulp and periodontal tissue cells. From a practical perspective, research on dental piezoelectricity offers new approaches and tools for the diagnosis and treatment of oral diseases. In terms of diagnosis, detecting changes in the piezoelectric response of teeth may enable early identification of pathologies such as dental caries and periodontitis, thereby improving diagnostic accuracy. In treatment, leveraging the principles of piezoelectricity offers opportunities to develop novel oral treatment technologies—for example, in teeth whitening and orthodontics—providing patients with safer and more effective therapeutic options. Furthermore, the study of teeth piezoelectricity has profound implications for both basic research and clinical practice in dentistry. On the basic research front, it stimulates in-depth exploration of bioelectrical phenomena in teeth and promotes interdisciplinary integration across fields such as oral biology and materials science. Clinically, piezoelectricity-based findings are expected to facilitate the development of more personalized and precise treatment methods, enhancing therapeutic outcomes and patients’ quality of life. Therefore, further investigation into the biological functions of teeth piezoelectricity is of great importance for advancing oral medicine.

Despite the significant advances achieved so far, hurdles exist in translating piezoelectric materials from bench to bedside. First, widespread clinical application imposes more stringent requirements on piezoelectric materials, including biocompatibility, significant piezoelectric properties, a wide range of material sources, and low cost. For injectable piezo-gel, biodegradability is also essential to avoid triggering inflammatory responses in vivo. Secondly, at the device level, repeatability and excellent long-term stability are essential—especially in scenarios where the device needs to be worn for extended periods, such as in orthodontics. In addition, lightweight and flexible devices can reduce foreign-body sensation and improve patient compliance. Thirdly, in practical applications, the diagnostic and therapeutic outcomes across different age groups should be comprehensively evaluated, and the protocols should be adjusted accordingly to enhance accuracy and effectiveness. Finally, the clinical application and commercialization of the device must comply with relevant regulations.

## Figures and Tables

**Table 1:** Applications of piezoelectric materials in dentistry.

Application Fields	Key Materials	Piezoelectricity	Development Stage	Ref.
Tissue regeneration and repair	Strontium-doped biofilm	d_33_ = 14 pC/N	Preclinical	[64]
	BaTiO_3_ dental composite resins	d_33_ = 0.5–4.2 pC/N	Preclinical	[65]
	Piezoelectric hydrogels	Output voltage = 45.4 mV	Preclinical	[66]
	Nylon-11 nanoparticles	PFM amplitude ~10 mV	Preclinical	[67]
	VDF-TeFE copolymer	d_33_ = 4 pC/N	Preclinical	[68,69,70]
Antibacterial effects	PVDF/TiO_2_ nanotubes coating	/	Preclinical	[74]
	Metal/piezoelectric nanostructures	PFM amplitude with butterfly loop and phase hysteresis	Preclinical	[75]
	Zn-MSNs/K18-MMA/K18-Filler in dental resins	/	Preclinical	[81,82]
	Injectable PiezoGEL	Output voltage ≈ 10 mV	Preclinical	[85]
Teeth whitening	BaTiO_3_-based systems	PFM amplitude with butterfly hysteresis loop and 180° phase switching	Preclinical	[90]
	Z-scheme g-C_3_N_4__−__x_/Bi_2_O_3__−__γ_ heterostructure	/	Preclinical	[94]
Oral health monitoring	Piezoelectric units embedded in aligners	d_33_ = 200–400 pC/N	Preclinical	[19]
	DA/PVA/glycine piezoelectric film	d_33_ = 10.8 pC/N	Clinical	[99]
	Piezoelectric film sensors	/	Clinical	[18,102]

**Table 2:** Evidence from in vitro, in vivo, and clinical studies on piezoelectric dental materials.

Materials	Study Model	Trial Data (*p* < 0.05)	Functions	Ref.
Piezoelectric units embedded in aligners	In vivo Rat model with Ni-Ti spring fixation between molar and incisor	Orthodontic efficiency increase: 34% (young) and 164% (aged) rats	Real-time orthodontic monitoring	[19]
Strontium-doped biofilm	In vivo Canine dentin defect	A 3-fold enhancement in dentin regeneration over conventional film.	Dentin mineralization	[64]
BaTiO_3_ dental composite resins	In vivo SBF solution, under cyclic mechanical loading	11.6 ± 4.1 μm mineral layer (vs. ~5 μm in control)	Remineralization and self-repair	[65]
Piezoelectric hydrogels	In vivo Rat alveolar bone defect	63.40 ± 5.58% alveolar bone regeneration (vs. 44.15 ± 10.01% in control)	Periodontal tissue regeneration	[66]
Nylon-11 nanoparticles	In vitro DPSCs co-culture with nylon-11 nanoparticles	~3-fold upregulation of osteogenic markers compared to control	Alveolar bone repair	[67]
VDF-TeFE copolymer	In vivo Rat oral mucosa wound defect	Significantly smaller wound area (4.8 ± 2.1 mm^2^) than control group (8.2 ± 1.7 mm^2^)	Oral mucosal wound healing	[68,69,70]
PVDF/TiO_2_ nanotubes coating	In vitro Co-incubated with *S. aureus* and *E. coli*	Inhibition of bacterial adhesion: 30.4% (*S. aureus)* & ~61.9% (*E. coli*)	Prevents peri-implant infections	[74]
Metal/piezoelectric nanostructures	In vivo Rat subcutaneous *S. aureus*-piezoelectric implant infection model	With an in vivo antibacterial rate of 96.9%	Inhibits *S. aureus* adhesion	[75]
Zn-MSNs/K18-MMA/K18-Filler in dental resins	In vitro The resin surface was inoculated with *S. mutans*	Significantly fewer *S. mutans* (725,333 ± 162,578 CFUs) than control group (1,620,333 ± 577,037 CFUs)	Inhibits caries progression	[81,82]
Injectable PiezoGEL	In vivo Rat ligature-induced periodontitis	Higher bone volume (55%) vs. control (20%)	Reduces plaque-related periodontal diseases	[85]
BaTiO_3_-based systems	Ex vivo The extracted teeth	Whitening efficiency (ΔE): ~3-fold higher than control	Achieves efficient and non-destructive tooth whitening without using high-concentration peroxides	[90]
Z-scheme g-C_3_N_4__−__x_/Bi_2_O_3__−__γ_ heterostructure	Ex vivo The extracted teeth	Pigment degradation efficiency: 97.6% (vs. 25.4% in control)		[94]
DA/PVA/glycine piezoelectric film	Clinical trial Assembled into a waterproof force sensor and placed on teeth occlusal surfaces	Detecting four types of occlusal contact patterns	Monitoring of occlusal contact during orthodontic treatment	[99]
Piezoelectric film sensors	Clinical trial Nocturnal tooth-to-splint contact forces were recorded in home environment	Bruxism group exhibited a significantly longer event duration (27 s/h) compared to the control group (7.4 s/h)	Monitoring of bruxism and occlusal forces	[18,102]

Note: SBF = Simulated body fluid; DPSC = Dental pulp stem cell.

## Data Availability

No new data were created or analyzed in this study. Data sharing is not applicable to this article.

## Abbreviations

The following abbreviations are used in this manuscript:

ERK	Extracellular signal-regulated kinase
MAPK	Mitogen-activated protein kinase
cAMP	Cyclic adenosine monophosphate
PKA	Protein kinase A
VEGF	Vascular endothelial growth factor
FGF	Fibroblast growth factor
PI3K	Phosphatidylinositol 3-kinase
Akt	Protein kinase B
Runx2	Runt-related transcription factor 2
DSPP	Dentin sialophosphoprotein
DMP-1	Dentin matrix protein 1
ATP	Adenosine triphosphate
VDF-TeFE	Vinylidene fluoride and tetrafluoroethylene
PVDF	Polyvinylidene fluoride
ROS	Reactive oxygen species
Zn-MSNs	Zinc-doped mesoporous silica nanoparticles
K18-MMA	K18-methyl methacrylate
PiezoGEL	Piezoelectric hydrogel
GelMA	Gelatin methacryloyl
DA	Dopamine
PVA	Polyvinyl alcohol
PFM	Piezoresponse force microscopy
DPSCs	Dental pulp stem cells
SBF	Simulated Body Fluid
